# The effect of social media dependence on the perceived academic performance of university students in Cameroon: the role of psychological well-being and social anxiety

**DOI:** 10.3389/fpsyg.2025.1598724

**Published:** 2025-10-24

**Authors:** Yvette Dufola Jaff, Dilan Ciftci

**Affiliations:** Department of Communication and Media Studies, Institute of Graduate Studies and Research, Faculty of Communication, Cyprus International University, Nicosia, Cyprus

**Keywords:** social media, psychological well-being, sense of belonging, social anxiety, academic performance

## Abstract

Social media usage has increased, raising worries about its potential effects on individuals’ mental health. The present study aims to establish the relationship between social media dependence and academic performance and isolate how sense of belonging, and social anxiety mediate this effect. It offers an enhanced analysis of the phenomena by expanding on contributions provided by researchers from various areas in the literature. This research comprises a quantitative survey of 595 Cameroonian social media users to evaluate the suggested hypotheses. The results show that social media dependence generally has a beneficial indirect effect on perceived academic performance. The direct impact of social media dependence on perceived academic performance is negative but statistically insignificant. The findings shows that the significant positive influence of social media usage on the sense of belonging implies a positive impact of social media on Cameroonian students. However, the direct negative relationship between social media use and academic performance is not statistically significant and may be due to the fact that the benefits of social media use to the sample under investigation outweighs its negative effects. The findings have important policy implications. They suggest that higher education stakeholders in Cameroon should harness the potential of social media to enhance students’ sense of belonging and academic engagement. Policymakers could develop guidelines for integrating social media into learning environments in ways that support students’ psychological well-being and academic outcomes.

## 1 Introduction

Social media has become an integral part of daily life for individuals worldwide, with its usage increasing significantly in recent years. Defined as “websites and online technologies that foster relationships between users by allowing them to share knowledge, ideas, and interests” (Swar and Hameed, 2017; Leong et al., 2019; Kemp et al., 2020), social media platforms such as Facebook, Twitter, WhatsApp, Instagram, and TikTok have revolutionized communication and information dissemination. These platforms are popular among students, who use them for social interaction and academic purposes. The widespread adoption of smartphones has further intensified social media usage, creating a near-constant digital presence in users’ lives (Asemah et al., 2013). While the rapid growth of Information and Communications Technologies (ICTs) has enabled global connectivity and improved access to information, it has also raised concerns about the negative effects of excessive social media use, particularly concerning academic performance and psychological well-being.

Social media usage among students has been extensively studied, with studies largely focusing on its impact on academic performance. A plethora of studies have highlighted the negative consequences of social media and smartphone usage, particularly when these technologies divert students’ attention away from important academic tasks. Students often spend significant time on social media platforms, reducing the time dedicated to studying or attending classes (Giunchiglia et al., 2018; Lepp et al., 2015; Samaha and Hawi, 2016). This time mismanagement is compounded by the addictive nature of social media, as students may feel compelled to stay connected to avoid missing out on social interactions, a phenomenon commonly referred to as “fear of missing out” (Reer et al., 2019; Roberts and David, 2020). As a result, a growing body of evidence shows a negative correlation between social media use and academic performance, with frequent engagement on these platforms leading to lower grades and academic achievement (Giunchiglia et al., 2018).

However, the relationship between social media use and academic performance is not entirely straightforward. In addition to the direct effects of social media on students’ academic outcomes, psychological factors such as anxiety, and sense of belonging could play a crucial mediating role in this relationship. These psychological variables can either exacerbate or mitigate the impact of social media use on academic performance. For example, excessive social media use has been linked to heightened levels of social anxiety and stress, as students may experience feelings of inadequacy when comparing themselves to others or when they feel disconnected from their peers (Kircaburun et al., 2020; Jiao et al., 2017). This anxiety can negatively affect their academic engagement, reducing their focus on academic responsibilities.

In some scenarios, however, social media use has been shown to exert no effect on academic performance as other factors such as sports games and time management were shown to have a much more negative impact (Alwagait et al., 2015). Also, when used for academic purposes, social media did not exert any academic benefits (Lau, 2017). Social media platforms also offer positive psychological benefits. They provide opportunities for social support, fostering a sense of belonging and community among users. Studies have shown that social media can help students build and maintain social connections, improving their overall psychological well-being (David et al., 2018; Bano et al., 2019; Barbosa, 2024). For many students, social media offers a platform for identity formation, self-expression, and the development of a supportive social network, all of which contribute to better mental health. These positive effects on psychological well-being may indirectly enhance academic performance, as students who feel socially connected and supported are more likely to be engaged in their academic pursuits (Karikari et al., 2017). Given these complex dynamics, it is critical to examine how psychological factors mediate the relationship between social media usage and perceived academic performance.

While the direct impact of social media on academic outcomes has been well-documented, less attention has been paid to the indirect pathways through which psychological well-being, social anxiety, and sense of belonging influence this relationship. Understanding these mediating factors is essential for developing a more nuanced view of how social media use affects students’ academic success. By considering both the positive negative psychological effects, this study aims to offer a comprehensive analysis of the ways in which social media shapes perceived academic outcomes.

To bridge the gap in the literature, this study employs an innovative methodological approach that combines both behavioral and psychological data. Previous studies have employed actual academic performance data such as GPA and CGPA. Most of these studies isolate a negative effect of social media use on academic performance (Giunchiglia et al., 2018; Lau, 2017). Some of the studies however do not identify any relationship (Alwagait et al., 2015; AlFaris et al., 2018). In this study however, we employ perceived academic performance which is an individual’s self-assessment of their academic performance. Perceived Academic Performance (PAP) refers to a student’s self-reported evaluation of their academic progress, including elements such as grades, task completion, and understanding of course material. It reflects internal assessments of competence and motivation, and is particularly useful when psychological and emotional influences are central to the study. This makes PAP appropriate for research on social media and well-being, where subjective perceptions strongly influence learning outcomes. Perceived academic performance has been observed to align with actual academic performance (Ferla et al., 2010) and should be more sensitive to psychological factors. This study also expands on existing research by incorporating psychological factors as mediators in the relationship between social media usage and perceived academic performance. Specifically, we investigate how social anxiety and sense of belonging influence this relationship, building on prior work that has explored the effects of social media on students’ mental health (Schønning et al., 2020; Bekalu et al., 2019; Barbosa, 2024). By integrating these psychological variables into the analysis, this research aims to provide a more comprehensive understanding of the mechanisms through which social media use affects academic performance. Also, to the best of our knowledge, no other study has attempted to ascertain the direct and indirect effect of social media usage on perceived academic performance in a Sub-Saharan African country like Cameroon. This offers a more nuanced perspective on the social media and academic performance relationship. Differences in lived experience between European and African countries may bring about changes in the empirical relationship.

The results of this study will have significant implications for both educators and policymakers. As social media becomes increasingly integrated into students’ daily lives, it is essential to develop strategies that help students manage their social media use in ways that support, rather than hinder, their academic success. The present study offers insights into how psychological factors can either amplify or buffer the effects of social media, providing guidance for interventions that promote healthy social media use so as to mitigate its negative consequences.

Therefore, this study aims to fill existing gaps by investigating the direct and indirect effects of social media usage on perceived academic performance among university students in Cameroon. Specifically, we focus on the mediating roles of social anxiety and sense of belonging. This dual-pathway approach contributes to the understanding of how social and psychological mechanisms intertwine with academic engagement in the African context. The findings are expected to inform culturally sensitive interventions that promote well-being and academic achievement in digital learning environments.

The remainder of this paper is organized as follows: Section two presents a comprehensive review of the literature on social media usage, academic performance, and psychological factors, drawing from both psychological and educational research. Section three outlines the research methodology, including the use of survey instruments to capture social media usage and its impact on academic performance. Section four presents the findings, examining the direct and indirect effects of social media use on academic performance, with a focus on the mediating role of psychological well-being, social anxiety, and sense of belonging. Finally, Section five discusses the implications of the findings, offering recommendations for future research and practical strategies for educators and policymakers to enhance academic performance in the digital age.

## 2 Review of past studies

### 2.1 Social media use and sense of belonging

A sense of belonging reflects a psychological, emotional, or cognitive state (Hagerty et al., 1992). Zhao et al. (2012) link belonging to social capital, identifying connections, perceived similarities, and trust among members as key drivers. Lin et al. (2014) emphasize that sustained social media use strengthens belonging as an emotional response, while Xu and Li (2015) highlight its role in fostering online community engagement. Though critical to user behavior, the relationship between belonging and habit formation on social media remains underexplored. Belonging enhances commitment to online communities, encouraging reciprocity and repeated use, which may automate behaviors over time (Verplanken, 2006; Zhao et al., 2012).

Social media significantly impacts university students’ lifestyles and social experiences (Mastrodicasa and Metellus, 2013), with studies linking it to psychological well-being, self-esteem, social capital, life satisfaction, and offline interactions (Bargh and McKenna, 2004; Ellison et al., 2011; Gonzales and Hancock, 2011; Jacobsen and Forste, 2011; Mihailidis, 2014). However, negative effects include depression post-breakup, cyberbullying, social exclusion, and academic decline (Junco and Cotten, 2012; Marshall, 2012; Nixon, 2014). These outcomes depend on factors like time spent online, motivations for use, and interaction quality.

Rooted in psychological theory, belongingness reflects a fundamental human need for meaningful connections (Baumeister and Leary, 2017; Maslow, 1968). Unmet belongingness correlates with loneliness and detachment (Baumeister and Leary, 2017), while its presence enhances social bonds, appropriateness, and academic outcomes (Allen and Kern, 2017; Neel and Fuligni, 2013; Walton et al., 2012). Scales such as the Sense of Belonging Measure and Need to Belong Scale assess this construct (Hagerty and Patusky, 1995; Leary et al., 2013).

Active social media engagement (e.g., supportive interactions) fosters well-being through social capital, whereas passive use (e.g., scrolling) risks jealousy and reduced well-being (Chen et al., 2020; Verduyn et al., 2017). Personality traits and usage patterns further mediate these effects (Hall, 2018; Wong et al., 2020).

### 2.2 Social media use and students’ academic performance

Since the establishment of these social media networks, students’ academic performance has improved significantly, and several studies have verified that the cyberspace plays a vital role for students in higher education. Rifkin et al. (2009) identified four (4) primary benefits of new media use by students in higher education in their study: building connections, increasing learning motivation, offering personalized course content, and fostering knowledge and skills. Moreover, throughout the 21st century, social media has tremendously benefited in the ease of learning. It has been established that a greater percentage of learners, particularly those at the Ph.D. level, significantly use social media to improve their academic performance (Khan, 2017). However, excessive social media use has been shown to impede academic performance due to reasons ranging from poor time management and short attention span (Paul et al., 2012; Alwagait et al., 2015), video gaming and social media multi-tasking (Lau, 2017) as well as usage addiction (Giunchiglia et al., 2018).

### 2.3 Social media use and social anxiety

Individuals with social anxiety often engage more frequently in passive social media use due to fear of negative evaluation in face-to-face interactions, limiting meaningful connections (Alden and Taylor, 2004; Hoffman et al., 2021). Their preference for online communication stems from maladaptive social beliefs, enabling controlled self-presentation and reduced real-time social pressure (Caplan, 2005; Davis, 2013; Weidman et al., 2012). This avoidance is compounded by tendencies toward negative social comparisons, exacerbating feelings of isolation (Antony et al., 2005). While socially anxious individuals may use the internet to compensate for offline social deficits (Shepherd and Edelmann, 2005), studies indicate they perceive online platforms as safer for socialization (Erwin et al., 2004), though this may reinforce avoidance behaviors.

Meta-analyses reveal that extraverts use social media to expand social opportunities, whereas socially anxious individuals engage online to address shortcomings, yet fail to accrue social benefits (Cheng et al., 2019). Hofmann’s (2000, 2007) framework categorizes social anxiety into: (1) unrealistic social performance expectations, mitigated by asynchronous online interactions; (2) negative self-image and rumination, potentially reduced through curated online discourse; and (3) reliance on digital platforms to avoid in-person contact. Despite perceived control over self-presentation, excessive online engagement correlates with poorer well-being (Caplan et al., 2009; Kraut et al., 2002; Weidman et al., 2012).

Emerging literature underscores the significance of digital identity and coping mechanisms in moderating the effects of social anxiety (Bozkurt and Tu, 2016). Mosharrafa et al. (2024) highlighted how mental health mediates social media’s academic effects, with self-regulation acting as a protective factor. Digital identity construction, often through carefully curated profiles and interactions, may either alleviate or intensify anxiety symptoms depending on context and individual traits. Therefore, coping strategies such as mindfulness, time management, and selective media engagement have been proposed as effective interventions to manage the psychological risks of social media use.

Studies highlight problematic internet use (e.g., compulsive behavior, psychosocial decline) as linked to social anxiety, even after adjusting for general anxiety and depression (Lee and Stapinski, 2012). While online interactions may provide temporary comfort (Prizant-Passal et al., 2016), they often serve as avoidance tactics, perpetuating anxiety by reducing face-to-face exposure (Caplan, 2005; Carruthers et al., 2019). Mixed outcomes emerge: internet use fosters new connections but risks weaker offline bonds (Erwin et al., 2004), and while instant messaging alleviates depression in socially isolated adolescents, it does not directly reduce social anxiety (Selfhout et al., 2009).

Critically, studies conflate general internet use (e.g., browsing) with social interactions, obscuring whether social engagement online mitigates harm (Prizant-Passal et al., 2016). Erwin et al. (2004) note that socially anxious users report both benefits (e.g., social support) and drawbacks (e.g., avoidance), though the absence of self-focused attention metrics limits conclusions. Overall, evidence suggests online engagement often reinforces maladaptive behaviors, functioning as a safety habit that sustains anxiety rather than alleviating it.

### 2.4 Theoretical framework

Katz et al. (1974) established the Theory of Uses and Gratifications. The approach concentrates on the consumer or audience rather than the message itself, questioning “what people do with media” rather than “what media does to people” (Katz, 1959). It is assumed that audience members are not passive but actively participate in understanding and integrating media into their own life. The approach also makes the viewer accountable for selecting media that meets their requirements. According to the idea, people utilize media to satisfy particular desires. This notion would subsequently suggest that the media competes for audience pleasure with alternative sources of information (Katz et al., 1974). The examination of Uses and Gratifications moves attention and focus away from media creation and transmission and toward media consumption. It emphasizes the idea that audiences are not passive consumers of media material, but rather reflect it via their desires, beliefs, and so on (Anaeto et al., 2008). Media audiences are typically satisfied by three sources: media content, media exposure, and the social context of exposure (Blumler and Katz, 1974). According to Katz et al. (1974), the uses and gratifications approach comprises five components. The components are:

- The audience is viewed as active.
- In the mass communication process, the audience member takes the lead in linking gratification and media selection.
- The media competes with other sources of pleasure.
- In terms of methodology, many of the goals of mass media usage may be determined from data contributed by individual audience members.
- Although market inclinations are being examined on their terms, evaluative judgments on the cultural significance of mass media must be deferred.

The paradigm stresses the media’s consuming usefulness rather than its transmission capabilities.

### 2.5 Hypotheses development

#### 2.5.1 The relationship between social media use, sense of belonging and academic performance

Earlier research claimed that perhaps the link between the prevalence of past behavior with habit is a hyperbolic curve. Early repeats lead to higher gains in fluency than later exposures in the habit design process (Lally et al., 2010). In the framework of social media, a sense of belonging has been recognized as a durable as well as a long-lasting emotional element that supports word recognition through continued social media site usage patterns (Zhao et al., 2012; Lin et al., 2014; Xu and Li, 2015). When one significant sense of belonging is developed inside the social media sites, then the capacity of recurrence to encourage user behaviors might well be lessened. For instance, among the most popular social media platforms for human engagement including expression transmission is active social media. Social media platforms allow people to create online profiles, share knowledge, as well as establish groups of acquaintances from across the world, thus satisfying individuals’ desire for just a sense of belonging and allowing them to expand existing connections. Whenever people form deep strong connections with their Facebook friends, their Facebook usage patterns are difficult to break, although people may reduce their overall frequency of use (Cheung et al., 2011; Thadani and Cheung, 2011; Deng and Tavares, 2013). In an empathetic way, individuals who participate in a social media platform strive and sustain dedicated connections with loyalties throughout the imaginary network as a result of their time and resource commitment. Within the context of academic performance, it is generally the case that a higher sense of belonging is associated with better academic performance (Huang, 2022; Li and Singh, 2023). Based on the aforementioned assessment, we posit that;

*H_1_: There will be a positive relationship between social media usage and students’ sense of belonging.*

*H_2_: There will be a positive relationship between General Social belongingness and the perceived academic performance of students.*

H_1_ and H_2_ are supported by the U&G premise that media is used to build and maintain interpersonal relationships, satisfying the need for belonging.

##### 2.5.1.1 Relationship between social media usage and the social anxiety of students

Social media significantly influences societal dynamics, particularly among students, who often rely heavily on these platforms. While social anxiety can hinder personal development, it affects both genders and may drive individuals to use online communication as a compensatory mechanism. Social media enables global connectivity but also risks isolating users from face-to-face interactions, reducing opportunities to interpret nonverbal cues or build trust. Prolonged device usage among youth often replaces direct social engagement, which can heighten anxiety for those already socially anxious. However, online platforms may offer a controlled environment to mitigate anxiety by avoiding immediate physical reactions (e.g., blushing) and allowing time to craft responses (Fernandez et al., 2012; Caplan, 2005).

The Social Compensation Hypothesis posits that socially anxious individuals use online interactions to navigate social challenges (Fernandez et al., 2012). Caplan (2005) suggests the absence of nonverbal cues (e.g., facial expressions) in digital spaces grants users greater control, reducing anxiety. Similarly, McKenna and Bargh (2014) argue that online interactions bypass anxiety triggers like physical appearance, enabling easier socialization. Lee and Stapinski (2012) corroborate that online platforms foster perceived control, enhancing confidence. Yen et al. (2012) found lower anxiety during online engagement compared to face-to-face interactions, though socially anxious individuals still exhibited higher anxiety online than non-anxious peers.

Contrastingly, Carruthers et al. (2019) notes that platforms like Facebook may replicate real-life anxiety triggers, as users with social anxiety report negative thoughts and protective behaviors akin to offline interactions. This suggests social media’s benefits are context-dependent, with certain platforms amplifying rather than alleviating anxiety. While online communication is often preferred by socially anxious individuals, its efficacy varies across platforms and user experiences.

Social anxiety is also revealed to have a negative effect on academic performance via its effect on social ties (Brook and Willoughby, 2015) and social anxiety disorder (Vilaplana-Pérez et al., 2021) and avoidance (Przepiorka et al., 2021). To this end, we posit that;

*H_3_: There will be a negative relationship between Social Media usage and the social anxiety of students.*

*H_4_: There will be a negative relationship between the social anxiety of students and the perceived academic performance of students.*

H_3_ and H_4_ derive from the idea that media helps users cope with interpersonal discomforts like social anxiety—though such coping may not always result in reduced anxiety.

##### 2.5.1.2 The relationship between social media and students’ academic performance

According to Mehmood and Taswir (2013), social media enhances learning and must be investigated as knowledge entrepreneurs. For a considerable time, increased internet utilization has been a universal phenomenon. Adolescents, as well as youngsters, have particularly identified these websites as a means to communicate with their classmates, share information, reinvent their selves, and promote their social life. Ellison et al. (2007). With the technological advancement that makes it easier to connect with people, as well as the awareness of the online platform, internet sites have evolved into mostly online activity, with Websites (Coyle and Vaughn, 2008). According to Astatke et al. (2023), social media users oftentimes experience poor performance academically.

Furthermore, Englander et al. (2010) contend that social media is inversely related to overall student academic achievement and is substantially more significant than its benefits. Internet addiction has resulted in an increase in internet activity over the previous several decades. Thus, according to Nalwa and Anand (2003), committed individuals choose to consume online content above the execution of their individual and professional duties, which eventually leads to poor academic performance. Along the same line, Karpinski et al. (2013) found that social media users spent less time on university academics than non - participants, resulting in a poorer Grade point average (GPA). It was also stated that, among the different distinctive diversions of each age, social media constitutes a prominent distraction of the modern era generation. We posit that;

*H_5_: There will be a negative relationship between Social Media usage and the academic performance of students.*

H_5_ reflects concerns with time displacement and gratification from non-academic use of social media.

##### 2.5.1.3 The indirect relationship between social media use and perceived academic performance

So far, the hypotheses proposed all postulate direct relationships between social media use and psychological factors; social media use and perceived academic performance as well as psychological factors and perceived academic performance. However, very little is known about how these psychological factors mediate the relationship between social media use and perceived academic performance. The direct relationship between social media use and psychological factors is well elucidated in the literature. Also, psychological factors have been shown to have a direct effect on academic performance. One of the ways through which psychological factors can enhance academic performance is digital identity formation in the social media space which enhances their social presence without necessarily impacting their physical space (Bozkurt and Tu, 2016). This further brings out the importance of isolating the latent mediation effects between social media use and perceived academic performance. To this end, we posit that;

*H_6_: The relationship between social media usage and perceived academic performance is positively mediated by the general social belongingness of students.*

*H_7_: The relationship between social media usage and perceived academic performance is negatively mediated by the social anxiety of students.*

**Figure 1:**
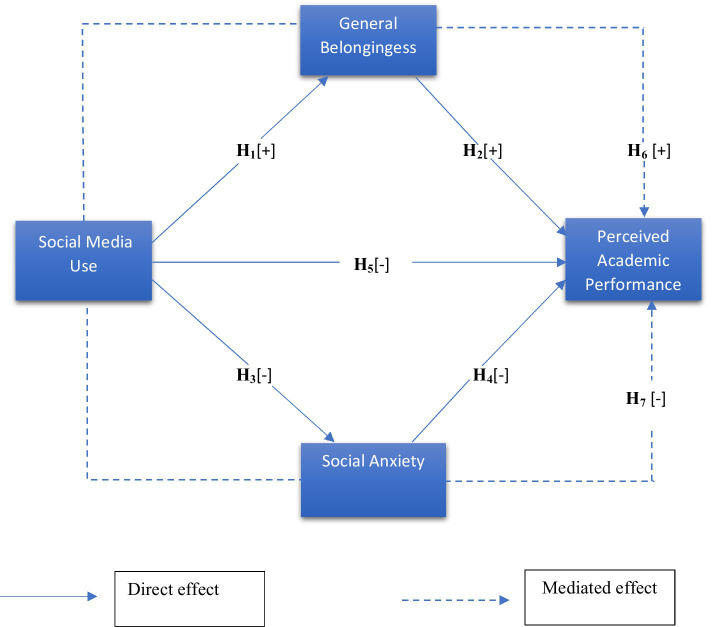
Research model.

H_6_ and H_7_ integrate these psychological mechanisms to show how gratification (belonging and anxiety reduction) can increase confidence in an individual’s academic perceptions (see Figure 1).

### 2.6 Summary of hypotheses

*H_1_: There will be a positive relationship between social media usage and students’ sense of belonging.*

*H_2_: There will be a positive relationship between General Social belongingness and the perceived academic performance of students.*

*H_3_: There will be a negative relationship between Social Media usage and the social anxiety of students.*

*H_4_: There will be a negative relationship between the social anxiety of students and the perceived academic performance of students.*

*H_5_: There will be a negative relationship between Social Media usage and the perceived academic performance of students.*

*H_6_: The relationship between social media usage and perceived academic performance is positively mediated by the general social belongingness of students.*

*H_7_: The relationship between social media usage and perceived academic performance is negatively mediated by the social anxiety of students.*

## 3 Methodology

### 3.1 Sample and data collection

Data collection for this study was conducted by distributing a survey link via email, mobile phones, and various social media platforms. Prior to completing the closed-ended questionnaire, participants were informed that their participation was voluntary, confidential, and anonymous. The data collection period spanned from February 6, 2022, to May 4, 2022, coinciding with the ongoing COVID-19 pandemic in Cameroon. It is important to note that the pandemic may have influenced the study results.

Participants representing various Cameroonian institutions were randomly selected. The selection criteria were as follows: (1) university students were considered an ideal sample for e-commerce research, particularly within the context of social media, (2) university students are known to be heavy users of mobile devices and, as research suggests, are prone to addiction (Mou et al., 2024; Zhao, 2023), and (3) the participants were well-educated, competent, and aware of the potential disadvantages of social media and excessive mobile phone use.

Due to the constraints of the COVID-19 pandemic, as well as financial and logistical limitations, a total sample size of 606 respondents was achieved using a convenience random sampling method. Additionally, quantitative empirical research was employed to assess the research model, with data collected through an online survey. This method was selected for two key reasons: the widespread impact of the pandemic and the need to reach a large number of participants. Online surveys are recognized as a robust and reliable tool in contemporary research (Dutot and Bergeron, 2016; Fan et al., 2021; Qalati et al., 2021), providing a quick, efficient, and cost-effective means of data collection.

The research utilized a well-designed, self-developed questionnaire titled “The Impact of Social Media on Students’ Psychological Well-Being, Sense of Belonging, Social Anxiety, and Academic Achievement.” This instrument was designed to gather relevant data from participants and was divided into two sections:

- *Section A* collected participants’ demographic and personal information.
- *Section B* comprised a set of questions with response options based on a Likert scale: Strongly Agree (SA), Agree (A), Slightly Agree (SLA), Neutral (N), Slightly Disagree (SLD), Disagree (D), and Strongly Disagree (SD).

### 3.2 Data analysis

#### 3.2.1 Common method bias and non-response tests

Given the cross-sectional nature of this study, assessing common method variance was essential. Following Hair et al. (2017), the Harman single-factor test was employed, and the largest variance explained was 32.99%, which is below the 40% threshold. Therefore, common method variance is not considered a significant issue in this study. Furthermore, the results revealed no significant differences in the mean values of participants (*p* > 0.05), indicating that non-response bias was not a concern.

#### 3.2.2 Data screening and pre-analysis

The dataset was carefully screened for missing values, normality, and demographic characteristics. Three cases with missing values were addressed using the mean replacement method recommended by Hair et al. (2017) and implemented in SmartPLS. This approach is preferred over pairwise and listwise deletion as it maintains the sample size and preserves the mean values of the variables, ensuring consistency in the dataset. Table 1 presents the demographic data of the participants. Participants were predominantly aged 21–25 years (47.5%), female (60.4%), and spent 1–5 h daily on social media (73.4%). Most were postgraduate students (68.2%), reflecting a sample with high academic engagement.

**Table 1:** Demographic.

Variables		Frequency	Percent
Age	18–20 years	56	9.2
	21–25 years	288	47.5
	26–30 years	177	29.2
	31 & above	85	14.0
Gender	Male	250	39.6
	Female	356	60.4
Marital status	Divorced	45	7.4
	In a relationship	252	41.6
	Married	82	13.5
	Single	227	37.5
Level of study	Masters	252	41.6
	PhD	161	26.6
	Undergraduate	193	3
Hours spent on social media per day	1 h	64	4.3
	1–3 h	242	40.0
	3-5 h	203	33.4
	More than 5 h	97	16.0
Year of study	1–2 years	97	16.0
	2–3 years	178	29.4
	3–5 years	213	35.1
	More than 5 years	118	19.5

Source: Authors’ construction.

#### 3.2.3 Measurement model

According to Fornell and Larcker, (1981); Gefen et al. (2000) the internal consistency, convergent validity, and overall discriminant validity of the study’s constructs were evaluated. The internal consistencies of the constructs utilizing composite reliability (CR), as demonstrated in Table 1, were satisfactory, with values >0.7. Furthermore, the seven constructions’ Cronbach alpha values, which are all >0.7, show a high level of dependability Also, all loadings of reflective indicators were substantial. Similarly, variables with values greater than 0.5 demonstrated appropriate convergent reliability (see Tables 2, 3).

**Table 2:** Measurement model.

Variables	Items	Cronbach’s alpha	Rho-A	Composite reliability	AVE
Social media use (Karikari et al., 2017)		0.948	0.949	0.963	0.866
Social media is part of my everyday activity	SM1				
Social media has become part of my daily routine	SM2				
I would be sorry if social media shut down	SM3				
I feel out of touch when I have not logged onto social media for a while	SM4				
Social anxiety (Liebowitz, 1987; Alkis et al., 2017)		0.935	0.935	0.954	0.838
I feel anxious about the fact that others might find my actions awkward	SA1				
I find it difficult mixing comfortably with the people I work with.	SA2				
I tense up if I meet an acquaintance on the street.	SA3				
I feel tense if I am alone with just one person.	SA4				
I have difficulty talking with other people.	SA5				
I find it difficult to disagree with another’s point of view	SA6				
Perceived academic performance (Verner-Filion and Vallerand, 2016)		0.817	0.82	0.88	0.649
I meet the official performance requirements expected out of a student	AP1				
I adequately complete assigned duties	AP2				
I fulfill responsibilities specified in the course	AP3				
I perform tasks that are expected of me	AP4				
My performance is beyond demands	AP5				
General social belongingness (Malone et al., 2012; Leary et al., 2013; Baumeister and Leary, 2017)		0.957	0.959	0.962	0.719
I have close bonds with family and friends	SB1				
When I am with other people, I feel included	SB2				
I feel accepted by others	SB3				
I have a sense of belonging	SB4				
I have a place at the table with others	SB5				
I feel connected with others	SB6				
I feel like an outsider	SB7				
I feel as if people do not care about me, because I do not belong	SB8				
I feel distant during the holiday season	SB9				
I feel isolated from the rest of the world	SB10				
When I am with other people, I feel like a stranger	SB11				

**Table 3:** Validity.

Variables	1	3	4	5
Social anxiety	**0.93**			
Social belongingness	0.446	**0.915**		
Academic performance	0.219	0.067	**0.806**	
Social media use	0.296	0.248	0.315	**0.848**

Square roots of AVEs in bold on the diagonal.

## 4 Results

The findings of this study provide some interesting insights into the relationship between social media usage, psychological factors, and perceived academic performance among university students in Cameroon. The results indicate that social media dependence does not have a direct and significant effect on perceived academic performance. Social media’s effect on academic performance has been observed to be negative with weak statistical evidence when psychological factors are controlled for. This aligns with previous studies that found weak direct associations between social media usage and academic performance when other variables were controlled for Alwagait et al. (2015) and Lau (2017).

However, social media usage has a statistically significant and positive effect on the general belongingness of students. This in turn has a strong positive impact on perceived academic performance. This aligns with prior findings (Zhao et al., 2012; Lin et al., 2014; Verduyn et al., 2017) which infer that social media fosters the development of social capital and the perception of a sense of belonging in students who actively engage in social media networks. The positive psychological feedback from this may enhance motivation and student engagement.

This finding also corroborates Shahzad et al. (2025) as well as Zhang et al. (2024) where it was revealed that collaborative learning platforms positively mediates the relationship between social media usage and academic performance. On the other hand, social anxiety did not have a significant relationship with social media usage, contradicting studies that suggest socially anxious individuals are more likely to engage in passive social media usage (Caplan, 2005; Weidman et al., 2012). The economic situation in Cameroon might imply the existence of difficult economic constraints in the physical space that can impact upon social anxiety. This may bring about the necessity for self-regulation (Reinecke et al., 2022) in the social media space so as to mitigate the potential for social media induced social anxiety.

Social anxiety was found to negatively impact perceived academic performance, reinforcing the notion that heightened anxiety can lead to avoidance behaviors and reduced academic engagement (Antony et al., 2005; Hofmann, 2007).

Furthermore, the mediation analysis revealed that the sense of belonging significantly mediates the relationship between social media usage and perceived academic performance, whereas social anxiety did not exhibit a significant mediating effect. The non-significant mediating role of social anxiety presents an intriguing deviation from several past studies. This may reflect cultural dynamics in Cameroon, where communal values and social norms potentially buffer the academic impact of online avoidance behaviors.

These findings suggest that the negative impact of social media usage on student’s academic performance in Cameroon is not mediated by social anxiety. Social anxiety has been observed to emanate from highly stressful environments (Mosharrafa et al., 2024), the social media environment of Cameroonian students may not meet the criteria of a highly stressful environment. According to the Uses and Gratifications Theory, students actively seek out media platforms such as social media to fulfil specific psychological and social needs—such as the need for belonging, information, or anxiety reduction. In the context of Cameroonian university students, this theoretical lens suggests that their engagement with social media platforms is not passive but rather intentional and functional. Having the power to choose which content to consume would imply that students will not choose social media environments that will induce a negative psychological effect. They will be more inclined toward content that will induce positive gratifications. Also, the fact that social anxiety does not emanate from social media use will entail that Cameroonian students’ social anxiety triggers is outside the social media space. This could also be a reflection of usage patterns that are unique to Cameroonian students, such as frequent use of WhatsApp academic groups rather than image-based platforms that promote social comparison. Moreover, certain unique cultural values in Cameroon may foster offline peer support structures that diminish the need for social validation online. The COVID-19 pandemic may also have reduced real-life social pressures, thus moderating the expected link between anxiety and digital engagement. One of the social innovations of the COVID-19 pandemic is the utilization of online educational facilities to enhance social distancing. Since social interactions were greatly diminished during this time, the utilization of social media for interaction and academic purposes may induce a greater sense of belonging in the psyche of Cameroonian students. Social media use may act as a coping mechanism for the stress induced by social distancing (Eden et al., 2020) (see Table 4).

**Table 4:** Hypotheses testing.

Hypotheses		*B*	Decision
H_1_	There will be a positive relationship between Social Media usage and the general social belonging of students.	0.52***	Supported
H_2_	There will be a positive relationship between General Social belongingness and the perceived academic performance of students	1.65***	Supported
H_3_	There will be a positive relationship between Social Media usage and the social anxiety of students.	0.01	Not supported
H_4_	There will be a negative relationship between the social anxiety of students and the perceived academic performance of students	−0.98***	Supported
H_5_	There will be a negative relationship between Social Media usage and the perceived academic performance of students	−0.47	Not Supported
H_6_	The relationship between social media usage and perceived academic performance is positively mediated by the general social belongingness of students	0.84***	Supported
H_7_	The relationship between social media usage and perceived academic performance is negatively mediated by the social anxiety of students	−0.04	Not significant

“***” Denotes that the *p* values are < 0.01.

## 5 Conclusion and implications

This study provides empirical evidence on the indirect effects of social media usage on perceived academic performance by incorporating psychological factors as mediators. While direct social media dependence does not significantly impact academic outcomes, the sense of belonging plays a crucial mediating role, reinforcing the idea that social integration fosters academic motivation and success.

The observed relationships, especially the strong link between sense of belonging and perceived academic performance, may have been influenced by the COVID-19 pandemic. During lockdowns and transitions to online learning, students in Cameroon may have relied more heavily on social media for peer support, which heightened the role of digital platforms in fulfilling social needs. Similarly, reduced in-person interactions might have lessened typical social anxiety triggers, explaining the non-significant link between social media usage and social anxiety. These outcomes suggest the findings are shaped by the unique conditions of the pandemic period.

Contrary to some prior studies (Brook and Willoughby, 2015; Vilaplana-Pérez et al., 2021), social anxiety did not emerge as a significant mediator, indicating that its impact on academic performance may be more complex and influenced by other external factors such as coping mechanisms and personality traits. These findings underscore the importance of fostering a healthy digital environment where students can leverage social media for social and academic benefits while mitigating potential stressors.

The results have important implications for educators and policymakers. Universities should encourage constructive social media use that enhances social belonging, such as peer support groups and academic networking opportunities. At the same time, interventions should be designed to support students with high social anxiety to ensure they do not disengage from academic responsibilities.

Based on the findings, Cameroonian universities should prioritize interventions that strengthen students’ sense of belonging through low-cost, culturally resonant strategies. For instance, institutions can promote WhatsApp-based peer learning groups, mentorship programs, and virtual campus events to create a stronger sense of academic community. These platforms are already familiar and widely used by students, requiring minimal investment. Additionally, academic advisors can collaborate with student groups to organize online discussion forums that support academic sharing and reduce isolation. Universities should also develop simple awareness campaigns that help students distinguish between helpful and harmful social media habits. Emphasizing academic use of social media over passive entertainment can help reduce time displacement and stress. In resource-constrained settings, leveraging existing technologies and student-led initiatives may be more sustainable than top-down interventions.

Educators should recognize the dual role of social media: fostering connectivity while necessitating strategies to mitigate time displacement. Interventions promoting active social media use (e.g., academic collaboration) over passive consumption could enhance belongingness without compromising academics. Furthermore, mental health programs targeting social anxiety may improve academic outcomes, even if unrelated to social media habits.

### 5.1 Limitations and future research

Self-reported data and a Cameroonian sample can limit generalizability. As such, replication in diverse contexts is needed. Differentiating active/passive social media use and exploring additional mediators (e.g., self-regulation) could deepen insights in future studies. The study reveals that social media’s impact on academic performance is mediated by psychological well-being, emphasizing the need for holistic approaches that balance digital engagement with mental health support. Additionally, this study treats social media usage as a unidimensional construct. However, prior research has differentiated between interactive use (e.g., commenting, sharing) and consumptive use (e.g., passive scrolling), which may yield divergent psychological and academic outcomes. Future research should disaggregate these modes of usage to clarify their distinct influences on well-being and academic success.

Future research should explore these relationships in different cultural and institutional contexts to validate these findings and further investigate potential moderating variables, such as personality traits and time management skills. Additionally, longitudinal studies would be beneficial in assessing the long-term impact of social media usage patterns on academic performance and psychological well-being.

## Data Availability

The raw data supporting the conclusions of this article will be made available by the authors, without undue reservation.
